# Gray Matter Variation in the Posterior Superior Temporal Gyrus Is Associated with Polymorphisms in the *KIAA0319* Gene in Chimpanzees (*Pan troglodytes*)

**DOI:** 10.1523/ENEURO.0169-21.2021

**Published:** 2021-12-14

**Authors:** William D. Hopkins, Nicky Staes, Michele M. Mulholland, Steven J. Schapiro, Madeleine Rosenstein, Cheryl Stimpson, Brenda J. Bradley, Chet C. Sherwood

**Affiliations:** 1 Department of Comparative Medicine, Michale E Keeling Center for Comparative Medicine and Research, The University of Texas MD Anderson Cancer Center, Bastrop, TX 78602; 2 Department of Anthropology and Center for the Advanced Study of Human Paleobiology, The George Washington University, Washington, DC 20052; 3 Behavioural Ecology and Ecophysiology Group, Department of Biology, University of Antwerp, Wilrijk, Belgium 2000; 4 Centre for Research and Conservation, Royal Zoological Society of Antwerp, Antwerp, Belgium 2000; 5 Department of Experimental Medicine, University of Copenhagen, Copenhagen, Denmark 1017; 6 Department of Pathology, Uniformed Services University, Bethesda, MD 20817; 7 Henry M. Jackson Foundation for the Advancement of Military Medicine, Bethesda, MD 20817

**Keywords:** chimpanzees, communication, gray matter, KIAA0319, language, neuropil fraction

## Abstract

Determining the impact that the *KIAA0319* gene has on primate brain morphology can provide insight into the evolution of human cognition and language systems. Here, we tested whether polymorphisms in *KIAA0319* in chimpanzees account for gray matter volumetric variation in brain regions implicated in language and communication (particularly within the posterior superior temporal gyrus and inferior frontal gyrus). First, we identified the nature and frequencies of single nucleotide variants (SNVs) in *KIAA0319* in a sample of unrelated chimpanzees (*Pan troglodytes* spp.). Next, we genotyped a subset of SNVs (those important for gene regulation or likely to alter protein structure/function) in a sample of chimpanzees for which *in vivo* T1-structural magnetic resonance imaging scans had been obtained. We then used source-based morphometry (SBM) to test for whole-brain gray matter covariation differences between chimpanzees with different *KIAA0319* alleles. Finally, using histologic sections of 15 postmortem chimpanzee brains, we analyzed microstructural variation related to *KIAA0319* polymorphisms in the posterior superior temporal cortex. We found that the SNVs were associated with variation in gray matter within several brain regions, including the posterior superior temporal gyrus (a region associated with language comprehension and production in humans). The microstructure analysis further revealed hemispheric differences in neuropil fraction, indicating that *KIAA0319* expression may be involved in regulation of processes related to the formation and maintenance of synapses, dendrites, or axons within regions associated with communication.

## Significance Statement

Studying the impact of language-related genes on primate brain morphology can provide insight into the evolution of human cognition and language systems. Here, we show that two *KIAA0319* variants in chimpanzees are linked to differences in gray matter volume of the posterior superior temporal gyrus, a region associated with language comprehension and production in humans. Examination of the microstructure of this region showed that one *KIAA0319* variant is associated with hemispheric differences in neuropil fraction, indicating that *KIAA0319* expression may be involved in the formation and maintenance of synapses, dendrites, or axons within a region directly involved in communication. Together, these findings suggest that *KIAA0319* variants may underlie individual variation in auditory processing and associated brain regions in nonhuman primates.

## Introduction

Characterizing the behavioral, neural and genomic systems that distinguish humans from other primates has been a central focus of comparative research. In humans, there are several key connected brain regions implicated in the production and comprehension of language, particularly within the left hemisphere, including the basal ganglia, supplemental motor, inferior frontal, and posterior superior temporal gyri (Goulven and Tzourio-mazoyer, 2004; Friederici, 2015; Skeide et al., 2016). Further, studies have discovered several genes that account for individual and phylogenetic variation in linguistic functions and their underlying neural systems (Fisher and Marcus, 2006; Fisher and Scharff, 2009; Preuss, 2012; Pfenning et al., 2014). Arguably, the most well-known so-called language gene is *FOXP2* (Lai et al., 2001; Enard et al., 2002). Individuals with a *FOXP2* R553H missense mutation exhibit oro-facial dyspraxia and concomitant decrements in gray matter volume in the basal ganglia, supplemental motor area, anterior cingulate, and inferior frontal gyrus (Vargha-Khadem et al., 2005).

The gene *KIAA0319* has similarly been implicated in language functions of humans. *KIAA0319* polymorphisms have been repeatedly associated with developmental dyslexia, a reading disorder marked by difficulties learning associations between sounds with letters and/or written words (Cope et al., 2005; Dennis et al., 2009; Scerri et al., 2011; Luciano et al., 2013; Sun et al., 2014). Meta-analyses have revealed that individuals with dyslexia show atypical patterns of gray matter volume, cortical thickness, and white matter connectivity in brain regions implicated in language (Eckert et al., 2016; Mascheretti et al., 2017). Furthermore, *KIAA0319* single nucleotide variants (SNVs) are associated with the prevalence of dyslexia and linked to differences in gray and white matter cortical organization, particularly within the posterior superior temporal lobe and parietal junction within the left hemisphere (Darki et al., 2012; Pinel et al., 2012; Jamadar et al., 2013; Eicher et al., 2016; Ozernov-Palchik and Gaab, 2016).

Studying the impact of variation in genes like *KIAA0319* on primate brain morphology can provide insight into the evolution of human cognition and language systems by clarifying the functional role of genetic variation in shaping neural circuits. To this end, we tested whether chimpanzee *KIAA0319* polymorphisms account for gray matter variation in brain regions implicated in language, particularly the posterior superior temporal and inferior frontal gyri. Comparative studies in nonhuman primates have shown that these regions play a role in the production and processing of species-specific communicative signals and vocalizations (Heffner and Heffner, 1984; Poremba et al., 2003, 2004; Gil-da-Costa et al., 2006; Petkov et al., 2008; Taglialatela et al., 2009). There is also evidence that chimpanzees, and to a lesser extent some monkeys, exhibit population-level morphologic and cellular asymmetries in the inferior frontal gyrus and superior temporal cortex, which overlap with Wernicke’s area in the human brain (Hopkins and Nir, 2010; Spocter et al., 2010; Lyn et al., 2011; Marie et al., 2018), one of the main regions influenced by *KIAA0319* polymorphisms. What remains unknown is whether individual variation in morphology or function in these brain regions is associated with *KIAA0319* polymorphisms in chimpanzees.

To test this hypothesis, we initially identified the frequencies of *KIAA0319* SNVs in a sample of unrelated chimpanzees. Next, we selected SNVs of interest for further genotype-phenotype study. We focused on SNVs present in the 5′ upstream region of the gene (likely important for gene regulation as they are in or near the promoter region and transcription start site of the gene), as well as nonsynonymous SNVs in the coding region of the gene (these cause amino acid changes and therefore are more likely to alter structure and/or function of the protein). Next, we genotyped these SNVs in a sample of chimpanzees for which *in vivo* T1-structural magnetic resonance imaging scans had previously been obtained. We used source-based morphometry (SBM) to examine whole-brain gray matter covariation differences across *KIAA0319* SNVs. Additionally, we analyzed microstructural variation related to *KIAA0319* polymorphisms in the posterior superior temporal cortex from histologic sections of postmortem chimpanzee brains.

## Materials and Methods

### Subjects

In the current study, we used archival structural T1-weighted magnetic resonance imaging scans obtained from 136 adult captive chimpanzees (79 females and 57 males). Captive chimpanzees in the United States descended from a founder population that was >95% from the western subspecies (*Pan troglodytes verus*; Ely et al., 2005). The subjects were socially housed at the National Center for Chimpanzee Care (NCCC), which is part of The University of Texas MD Anderson Cancer Center. The NCCC facilities have indoor/outdoor enclosures with climbing structures, bedding, and daily environmental enrichment (Neal Webb et al., 2018). All chimpanzees had 24-h access to both indoor and outdoor areas except during cleaning. Care staff fed the chimpanzees a diet of commercially available primate chow and fresh produce multiple times per day, as well as several daily foraging opportunities and *ad libitum* access to water. All work was approved by the Institutional Animal Care and Use Committee at NCCC, followed the guidelines of the Institute of Medicine on the use of chimpanzees in research, and complied with the Society for Neuroscience Policy on Ethics.

### Identifying SNVs

Initial quantification of genetic variation in chimpanzees was done using publicly available whole genome data [*P. troglodytes troglodytes N* = 18, *P. troglodytes verus N* = 12, *P. troglodytes schweinfurthii N* = 16, *P. troglodytes ellioti N* = 10, *P. troglodytes* (unknown subspecies) *N* = 3; Prado-Martinez et al., 2013; De Manuel et al., 2016]. The genomes were mapped to the human genome (version hg19) using BWA-MEM v0.7.5a-r405 (http://bio-bwa.sourceforge.net/bwa.shtml) with default parameters. After removing duplicates using PICARD v1.91 (https://sourceforge.net/projects/picard/files/picard-tools/1.91/), SNVs were called using GATK *UnifiedGenotyper* (https://software.broadinstitute.org/gatk/documentation/tooldocs/current/org_broadinstitute_gatk_tools_walkers_genotyper_UnifiedGenotyper.php). Gene consensus sequences were built for each individual using *vcfconsensus* (http://vcftools.sourceforge.net/man_latest.html). Intronic regions were removed from the sequences and remaining promoter region and exons were aligned to the human *KIAA0319* reference sequence (Ensembl: ENSG00000137261) using Geneious (version 6.0.6).

Next, to confirm the presence and allele frequencies of the genetic variants in the population of chimpanzees for which we have matching neuroanatomical data, we genotyped the SNVs that showed variation in genome data for *P. troglodytes verus* in an additional set of 25 presumed unrelated chimpanzees. Only variants that showed minor allele frequencies >5% were then genotyped for the complete population. To infer the putative functional consequences of the resulting coding variants, we used SNAP2 (Hecht et al., 2015), a trained classifier based on a machine-learning device called a “neural network.” It distinguishes between effect and neutral variants/nonsynonymous SNVs by taking a variety of sequence and variant features into account. The effect of a variant is believed to be of importance to the native protein function if the SNAP2 score exceeds 50, is neutral if the score is below −50 and is unreliable when between 50 and −50.

### DNA extraction and genotyping

For the chimpanzees with matching neuroanatomical data, genomic DNA was extracted from 200-μl blood samples using the QIAampDNA Mini kit automated on a QiaCube (QIAGEN), following the manufacturer’s instructions. The DNA extract was recovered in 200 μl of elution buffer and kept frozen at –20°C. Negative extraction controls showed no evidence of DNA contamination. DNA concentrations were quantified using a Nanodrop 2000 (Thermo-Fisher Scientific) spectrophotometer. Subsequent genotyping of the SNVs of interest was done using either Sanger sequencing or High-Resolution Melt Analysis (Smith et al., 2010). For exons where multiple SNVs were present in relatively short fragments (<500 bp), exons were amplified using PCR. Primers were developed flanking the region with the SNVs (exon 4, forward CTT GGA AAC CAC CAG AAT CAT GCG G and reverse CTT TCT CCA ACA CCT CTC CTG AAG; exon 18, forward CTC AGC CCT ACA CAC CTC TTT G and reverse AAG CCT CCT CTA TAC CAG AAC TC). The 25-μl PCR mix contained 10.5 μl MasterMix (QIAGEN; HotStarTaq plus DNA polymerase, deoxynucleotides, and MgCl_2_), 2.0 μl primer mix (10 mm concentration of combined forward and reverse primers) and ∼5–20 ng of genomic DNA (quantified via a NanoDrop microvolume spectrophotometer). PCR started with an initial activation at 95°C (5 min), followed by 35 cycles of denaturing at 95°C (10 s), annealing at 54°C (30 s) and extension at 72°C (1 min), with a final extension period of 10 min at 72°C. All reactions included positive and no-template controls. The amplicons were then Sanger sequenced on an Applied Biosystems platform following manufacturer’s specifications at the Yale DNA Analysis Facility. For <100-bp exon sequences where only one SNV site was present, High-Resolution Melt Analysis (as in Jacobs et al., 2016) was used to assess genotypes. Primer pairs flanking the SNVs were designed to target a short segment (∼100 bp) containing the single polymorphic site (chr6:24899739, forward CTG ACT GAG ACT GGG AAC and reverse CTG TGG TTT AGA TTA TCA CG; chr6:24900865, forward GCA ATT CGT GAG GTT GGG and reverse GCC CTG AGT TCA GAA GG; chr6:24828663, forward GAA TGG AAA CCA GAG CAG TG and reverse ATG GAG AAC TTG CCT GCA TGA). All qPCRs and melting curves were generated on a Rotor-Gene Q (QIAGEN) platform. The 25 μL qPCR mix contained 12.5 μL HRM MasterMix (QIAGEN; HotStarTaq plus DNA polymerase, EvaGreen dye, Q-Solution, deoxynucleotides, and MgCl_2_), 1.75 μl primer mix (10 mm concentration forward and reverse primers) and ∼20 ng of genomic DNA (quantified via a NanoDrop microvolume spectrophotometer). qPCR started with an initial activation at 95°C (5 min), followed by 40 cycles of denaturing at 95°C (10 s), annealing at 54°C (30 s) 72°C (40 s) and a final extension period of 10 min at 72°C. In each reaction, no-template controls were included. Data on melting curves and temperature of qPCR products were generated immediately following amplification by increasing temperatures from 65°C to 95°C, rising by 0.1°C/2 s. Fluorescence data were plotted as a function of temperature during DNA denaturation (melting) and visualized and compared using the Rotor-Gene Q HRM software package (QIAGEN). The resulting melting temperatures and curve shapes were assigned to different genotypes based on previously established reference curves (as in Jacobs et al., 2016) of known variants. Genotypes were confirmed using multiple independent qPCR and high-resolution melt analyses (mean = 2.76 replicates per sample). Additionally, for each melting curve-based genotype, we Sanger sequenced 10% of the samples to validate and confirm the genotype (Applied Biosystems Genetic Analyzer, DNA Analysis Facility at Yale University). Multiple alignments of the resulting DNA sequences were performed using Geneious (version 6.0.6).

### Magnetic resonance image acquisition

We previously obtained magnetic resonance images using methods described elsewhere (Bogart et al., 2014; Bard and Hopkins, 2018; Mulholland et al., 2020). No new magnetic resonance images were collected for this study. Briefly, subjects were initially immobilized by injection of ketamine (10 mg/kg) or telazol (2–6 mg/kg) and subsequently anesthetized with propofol (40–60 mg/kg) per standard institutional guidelines. The subjects were then transported to the imaging facility and remained anesthetized for the duration of the scan (40–60 min depending on brain size), as well as during transportation back to a recovery cage (∼75–120 min in total). The subjects were placed in the scanner chamber in a supine position with their head inside the human-head coil. The chimpanzees were scanned using a 1.5-Tesla scanner and T1-weighted images were collected in the transverse plane using a gradient echo protocol (pulse repetition = 19 ms, echo time = 8.5 ms, number of signals averaged = 8, and a 256 × 256 matrix). After completing the magnetic resonance imaging scan collection, the subjects were temporarily housed in a single cage for several hours to allow them to recover from the anesthesia, after which they were returned to their social group.

### Postimage processing and analyses

The archival magnetic resonance imaging data were processed on a Macintosh computer using previously described methods (Mulholland et al., 2020). First, we imported the raw DICOM files into 3D Slicer 4 (www.slicer.org) and converted each into NIfTI format (Fedorov et al., 2012; Kikinis et al., 2014). Next, we used the BET function in FSL for skull-stripping, using fractional intensity thresholds ranging between 0.35 and 0.80 (Smith, 2002; Jenkinson et al., 2005). The skull-stripped brains were then imported into 3D Slicer for N4ITK bias correction (spline distance = 50, bias field = 0.15, convergent threshold = 0.001; Boyes et al., 2008; Tustison and Gee, 2009; Tustison et al., 2010, 2014). Using the MRI Denoising Package for MATLAB (R2015b; MathWorks), the scans were then denoised using an optimized nonlocal means denoising filter (Coupe et al., 2008). The scans were then resampled at 0.625 isotropic voxels and aligned in radiologic space in Analyze 11.0 (AnalyzeDirect), using a capsule placed during the imaging process as a left-right orientation marker. Finally, we used the FLIRT function in FSL to perform a 12-parameter affine linear registration (Jenkinson and Smith, 2001; Jenkinson et al., 2002) of the processed scan to a chimpanzee template brain (Hopkins and Avants, 2013).

We used the FSL-VBM pipeline (http://fsl.fmrib.ox.ac.uk/fsl/fslwiki/FSLVBM) to process each preprocessed scan. The FSL-VBM pipeline included (1) segmentation of each scan into gray and white matter, (2) linear registration of each scan to a standard chimpanzee template (Hopkins and Avants, 2013), (3) creation of a study-specific gray matter template (Smith et al., 2004; Andersson et al., 2007; Douaud et al., 2007), (4) nonlinear registration of each subject’s gray matter images to the study-specific template, (5) modulation of the gray matter volume by use of a Jacobian warp to correct for local expansion or contraction of gray matter within each voxel, and (6) smoothing with an isotropic Gaussian kernel with a σ of 2 mm.

### SBM and statistical analyses

Following previously described methods (Hopkins et al., 2019; Mulholland et al., 2020), we subjected the individual modulated gray matter volume produced in the FSL-VBM pipeline to perform SBM using the software program GIFT (https://trendscenter.org/software/gift/). SBM is a relatively new method used to characterize gray and white matter structural covariation in samples of magnetic resonance imaging scans (Alexander-Bloch et al., 2013; Bard and Hopkins, 2018). Unlike univariate analytic methods, such as voxel-based morphometry, SBM is a multivariate, data-driven analytic approach that utilizes information about relationships among voxels to group voxels carrying similar information across the brain. Without requiring prior determination of regions of interest, the resulting components or sources are identified based on the spatial information between voxels grouped in a natural manner and represent similar covariation networks between subjects; thus, this approach has been described as a multivariate version of voxel-based morphometry (Xu et al., 2009). At the individual level, GIFT outputs weighted scores for each component that reflect the relative contribution of each subject’s gray matter volume to their generation. For this sample of chimpanzees, the SBM analysis yielded eight components. The weighted scores for each of the eight components were the dependent measures in the subsequent multivariate analysis of covariance (MANCOVA). Sex and *KIAA0319* SNV alleles were the independent variables, while genetic relatedness was the covariate.

### Microstructural analyses of the posterior superior temporal cortex (area Tpt)

In addition to the *in vivo* SBM analysis, we selected area Tpt for histologic analysis because it overlaps with one of the larger gray matter regions that differed between chimpanzees in the SBM analysis. For histologic sectioning, tissue blocks were frozen on dry ice, and cut at 40 μm on a freezing sliding microtome. Every 10th section (spaced 400 μm apart) from each block was mounted on chromalum-subbed slides, stained using 0.5% cresyl violet to visualize cytoarchitecture, and coverslipped with DPX.

We quantified the neuropil fraction from the posterior superior temporal cortex (area Tpt) in the postmortem brains of 15 chimpanzees (ages 12–48 years, mean 32.6 years; seven female, eight male). The neuropil is defined as the space between neuronal and glial cell bodies, which is comprised of dendrites, axons, synapses, and microvasculature (Spocter et al., 2012). The neuropil fraction data came from previously published data in 12 individuals from Spocter et al. (2012), supplemented by an additional three brains measured for the current study, which had been processed more recently for histology. Neuropil fraction was measured using high-resolution images of these Nissl-stained sections. Imaging of the regions of interest was performed with a Zeiss Axioplan 2 photomicroscope (Zeiss) equipped with a Ludi XY motorized stage (Ludl Electronics), Heidenhain *z*-axis encoder, and an Optronics MicroFire color video camera coupled to a Dell PC using Stereoinvestigator software (MBF Bioscience). For each chimpanzee brain, three evenly spaced coronal sections were sampled throughout the region of interest. For each section, regions of interest were contoured under low magnification (2.5× objective lens) underneath a representative portion of the area (for cortical regions this was ∼3 mm in length along the cortical surface). At least 30 systematically random sampled images were taken within the contours of each section using a 20× objective lens, resulting in images at 0.53 pixels/μm resolution. Each image was imported into ImageJ (v.1.32j) and subjected to background subtraction with a rolling ball radius of 50 pixels and then converted to binary by an automated threshold routine (Spocter et al., 2012). Before calculation of the neuropil fraction, images that contained artifacts were removed from the batch and the remaining images were blind-coded to avoid observer bias.

We also analyzed previously published data on regional volume, total neuron number, and neuron density from area Tpt in 12 chimpanzees (Spocter et al., 2010). In brief, stereologic methods were used, including Cavalieri point counting to calculate the regional volumes of area Tpt, and optical fractionator sampling to estimate neuron numbers. In the cresyl violet Nissl-stained sections, neurons were distinguished from glial cells by the presence of a large, lightly stained nucleus and a distinct nucleolus, accompanied by lightly stained dendritic processes. Stereologic counts of neuron numbers based on cresyl violet Nissl-staining have been shown to produce results that are highly correlated with other methods of labeling neurons, such as NeuN immunostaining (Zhu et al., 2015).

We ran a series of multivariate general linear models using Type III sum of squares with each dependent measure of cortical area Tpt neuropil fraction, regional volume, neuron number, and neuron density in the left and right hemisphere, with sex, age, and brain mass as covariates, and all three *KIAA0319* genotypes as fixed factors. We also analyzed the asymmetry quotient (AQ; right – left/average of hemispheres) of neuropil fraction using general linear models with sex, age, and brain mass as covariates.

## Results

### KIAA0319 genetic variation

We identified 33 SNVs in chimpanzee *KIAA0319* 5′ flanking region and coding regions combined (see Table 1). Out of 33 SNVs, four were in the upstream flanking region and 29 were in the amino acid coding region. A total of 18 out of 29 SNVs were nonsynonymous variants, and only two of these were present in *P. troglodytes verus*: chr6:24841281: C/A in exon 4 resulting in a Val295Phe substitution, and chr6:24828663: A/C in exon 10 resulting in a Glu563Asp substitution. Next, we genotyped these two SNVs in our population of chimpanzees with matching neuroanatomical data and found that only chr6:24828663: A/C showed minor allele frequencies >5% and was thus suitable for further genotype-phenotype analysis. Genotype frequencies for this SNV (hereafter referred to as *KIAA0391_*Glu563Asp) were in Hardy Weinberg equilibrium (χ^2^ = 0.52, df* *=* *1, *p *=* *0.47) and SNAP2 prediction of its functional consequences resulted in a modest SNAP2 effect score of 35 (expected accuracy 66%).

**Table 1:** Chimpanzee *KIAA0319* genetic variation

Exon	Coordinate	Substitution	AA (change)	Genotype distribution
Promoter	chr6:24899739	T/A	**/**	T/T: 61 (92.754%); T/A: 6 (8.696%); A/A: 2 (2.899%)
Promoter	chr6:24899741	C/G	**/**	C/C: 66 (95.652%); C/G: 3 (4.34%); G/G: 0 (0%)
Promoter	chr6:24899753	C/A	**/**	C/C: 59 (85.507%); C/A: 8 (11.594%); A/A: 2 (2.899%)
Promoter	chr6:24900865	A/G	**/**	A/A: 49 (71.014%); A/G: 8 (11.594%); G/G: 12 (17.391%)
Exon 3	chr6:24849382	G/A	Q/R	G/G: 67 (97.101%); G/A: 2 (2.899%); A/A: 0 (0.000%)
Exon 3	chr6:24849393	C/G	F/L	C/C: 35 (50.725%); C/G: 16 (23.188%); G/G: 18 (26.087%)
Exon 3	chr6:24849744	G/T	E/D	G/G: 67 (97.101%); G/T: 2 (2.899%); T/T: 0 (0.000%)
Exon 3	chr6:24849935	C/T	I/V	C/C: 67 (97.101%); C/T: 2 (2.899%); T/T: 0 (0.000%)
Exon 3	chr6:24849938	C/T	I/V	C/C: 51 (73.913%); C/T: 13 (18.841%); T/T: 5 (7.246%)
Exon 4	chr6:24841281	C/A	V/P	A/A: 20 (28.986%); A/C: 23 (33.333%); C/C: 26 (37.681%)
Exon 7	chr6:24833567	G/A	S	G/G: 63 (91.304%); G/A: 6 (8.696%); A/A: 0 (0.000%)
Exon 9	chr6:24830381	G/A	S/G	G/G: 66 (95.652%); G/A: 3 (4.348%); A/A: 0 (0.000%)
Exon 9	chr6:24830388	G/A	P	G/G: 64 (92.754%); G/A: 5 (7.246%); A/A: 0 (0.000%)
Exon 9	chr6:24830414	T/C	Q/*	T/T: 64 (92.754%); T/C: 3 (4.348%); C/C: 2 (2.899%)
Exon 9	chr6:24830427	G/A	K	G/G: 64 (92.754%); G/A: 5 (7.246%); A/A: 0 (0.000%)
Exon 10	chr6:24828663	A/C	E/D	A/A: 31 (44.928%); A/C: 25 (36.232%); C/C: 13 (18.841%)
Exon 10	chr6:24828780	A/G	A	A/A: 66 (95.652%); A/G: 3 (4.348%); G/G: 0 (0.000%)
Exon 10	chr6:24828803	C/T	P/S	C/C: 68 (98.551%); C/T: 1 (1.449%); T/T: 0 (0.000%)
Exon 12	chr6:24822174	G/A	R	G/G: 67 (97.101%); G/A: 2 (2.899%); A/A: 0 (0.000%)
Exon 13	chr6:24821029	T/C	T	T/T: 59 (85.507%); T/C: 0 (0.000%); C/C: 10 (14.493%)
Exon 13	chr6:24821039	C/T	P/S	C/C: 57 (82.609%); C/T: 2 (2.899%); T/T: 10 (14.493%)
Exon 14	chr6:24818894	C/T	T/M	C/C: 67 (97.101%); C/T: 2 (2.899%); T/T: 0 (0.000%)
Exon 15	chr6:24816560	G/A	Q	G/G: 66 (95.652%); G/A: 2 (2.899%); A/A: 1 (1.449%)
Exon 17	chr6:24811335	C/T	V	C/C: 41 (59.420%); C/T: 23 (33.333%); T/T: 5 (7.246%)
Exon 17	chr6:24811348	T/C	T/I	T/T: 65 (94.203%); T/C: 4 (5.797%); C/C: 0 (0.000%)
Exon 17	chr6:24811366	G/A	Q/R	G/G: 59 (85.507%); G/A: 0 (0.000%); A/A: 10 (14.493%)
Exon 17	chr6:24811377	T/C	V	T/T: 66 (95.652%); T/C: 2 (2.899%); C/C: 1 (1.449%)
Exon 18	chr6:24808849	G/A	K	G/G: 65 (94.203%); G/A: 4 (5.797%); A/A: 0 (0.000%)
Exon 18	chr6:24808881	T/G	V/L	T/T: 65 (94.203%); T/G: 4 (5.797%); G/G: 0 (0.000%)
Exon 18	chr6:24808954	C/T	K/N	C/C: 66 (95.652%); C/T: 2 (2.899%); T/T: 1 (1.449%)
Exon 18	chr6:24808962	G/C	L/V	G/G: 61 (88.406%); G/C: 6 (8.696%); C/C: 2 (2.899%)
Exon 20	chr6:24803711	C/G	S/*	C/C: 43 (62.319%); C/G: 19 (27.536%); G/G: 7 (10.145%)
Exon 20	chr6:24803770	G/T	S	G/G: 25 (36.232%); G/T: 16 (23.188%); T/T: 28 (40.580%)

Coordinates are shown in reference to Pantro 4 (UCSC genome browser).

Out of the four SNVs present in the promoter flanking region, two were in complete linkage disequilibrium in our chimpanzee population (chr6:24899739:T/A and chr6:24899753:C/A), therefore only one was included in further genotype-phenotype analysis. A third SNV (chr6:24899741:C/G) did not exceed the >5% minor allele threshold and was also excluded from further analysis. For the two promoter region flanking region SNVs that were included in the analysis, genotype frequencies were in Hardy Weinberg equilibrium (chr6:24899739:T/A χ^2^ = 3.79, df* *=* *1, *p *=* *0.05; chr6:24900865:A/G χ^2^ = 2.84, df* *=* *1, *p *=* *0.09; hereafter referred to as *KIAA0391rsP1* and *KIAA0391rsP4*, respectively). The specific SNVs used in the subsequent analyses are highlighted in Table 1.

### KIAA0319 and gray matter covariation

**Figure 1.**
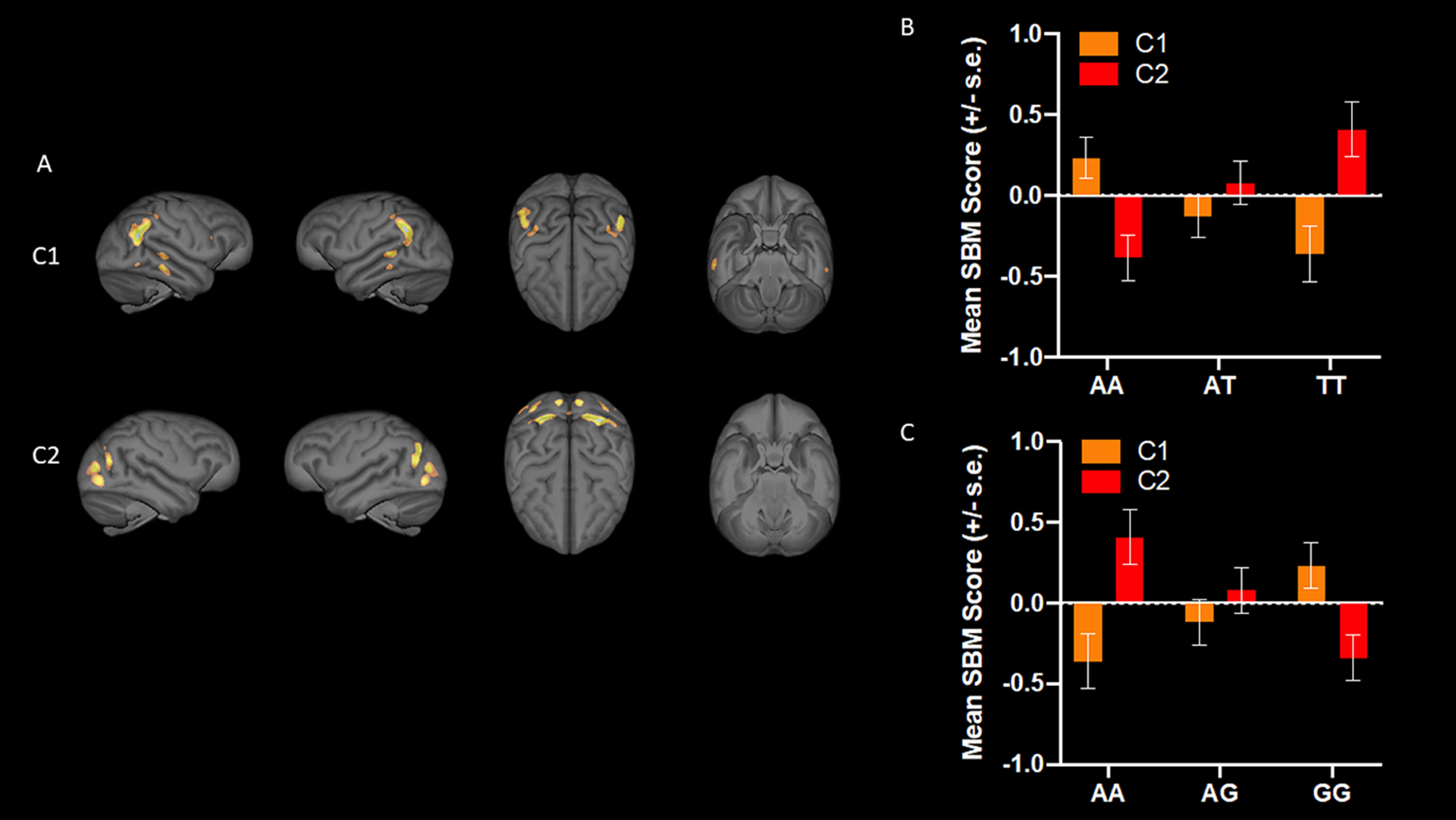
***A***, 3D renderings of SBM C1 and C2 (from left to right: right, left, top, and bottom views), and mean (±SE) SBM weighted scores for each allele of (***B***) *KIAA0319rsP1* and (***C***) *KIAA0319rsP4*. ***B***, Chimpanzees with the AA allele had higher weighted SBM scores on C1 compared with AT and TT individuals but the AT and TT means did not differ significantly from each other. For component two, AA, AT, and TT groups all differed significantly from each other. ***C***, For *KIAA0319rsP4,* chimpanzees with the minor AA allele had lower weighted SBM scores for C1 compared with GG but not GT individuals. By contrast, for C2, chimpanzees with AA had significantly higher values than GG but did not differ from AG chimpanzees.

**Figure 2.**
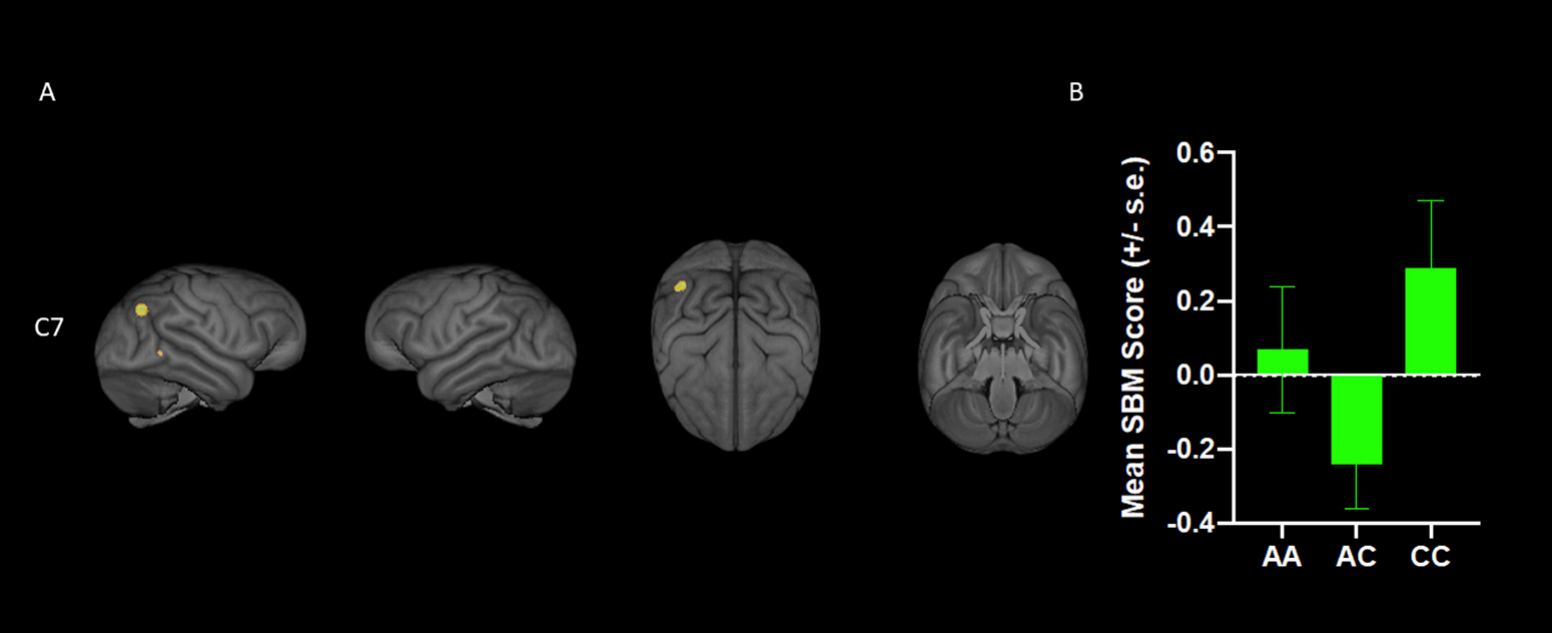
***A***, 3D renderings of SBM component 7 (from left to right: right, left, top, and bottom views). ***B***, Mean (±SE) SBM weighted scores for each allele of the *KIAA0319 Glu563Asp* variant. Chimpanzees with the major AC allele had lower weighted SBM scores for component 7 than individuals with the CC but not AA allele.

**Figure 3.**
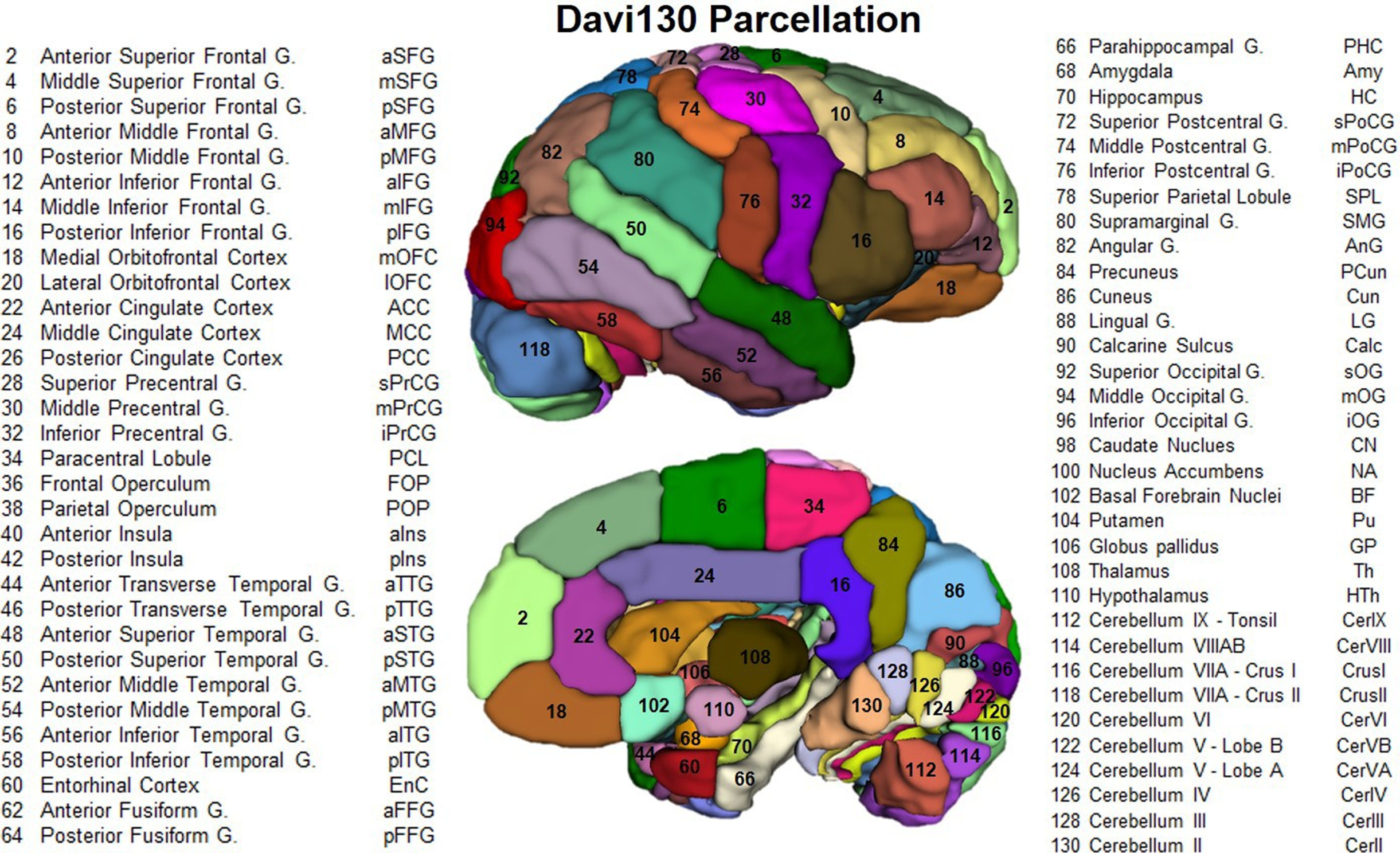
Lateral and medial views of the right hemisphere Davi130 parcellation of the chimpanzee brain, region numbers correspond to names in the figure. Reprinted with permission (Vickery et al., 2020).

For *KIAA0319rsP1*, the MANCOVA revealed a significant main effect for genotype *F*_(16,246)_ = 2.086, *p *= 0.01. Subsequent univariate *F*-tests revealed significant differences between the alleles for SBM component 1 (C1) *F*_(2,129)_ = 4.186, *p *=* *0.017 and component 2 (C2) *F*_(2,129)_ = 6.394, *p *=* *0.002 (Fig. 1*A*). *Post hoc* analysis for C1 indicated that chimpanzees with the AA allele had higher weighted scores compared with AT and TT individuals, but the AT and TT means did not differ significantly from each other. For C2, *post hoc* analysis indicated that the means for the AA, AT, and TT groups all differed significantly from each other (Fig. 1*B*). No other SBM components differed across the *KIAA0319rsP1* alleles. For *KIAA0319rsP4*, the results were very similar. The MANCOVA revealed a significant main effect for *KIAA0319* genotype *F*_(16,244)_ = 2.790, *p *= 0.007. Univariate *F*-tests revealed significant main effects of the SNV genotype on C1 *F*_(2,128)_ = 3.810, *p *= 0.025 and C2 *F*_(2,128)_ = 5.884, *p *= 0.004 (Fig. 1*A*). *Post hoc* analysis for component C1 indicated that chimpanzees with the minor AA allele had lower weighted scores compared with GG, but not GT individuals. By contrast, for C2, *post hoc* analysis indicated that AA had significantly higher values than GG but did not differ from AG apes (Fig. 1*C*). No other SBM components differed across the *KIAA0319rsP4* alleles. For the *KIAA0319 Glu563Asp* variant, the MANCOVA revealed no significant main effects or interactions. Notwithstanding, the univariate *F*-tests revealed a significant main effect for the genotype on C7 *F*_(2,1298)_ = 3.980, *p *= 0.046 (Fig. 2*A*). Chimpanzees with the major AC allele had lower weighted scores than individuals with the CC but not AA allele (Fig. 2*B*). No other SBM components differed across the *KIAA0319 Glu563Asp* alleles. (Note, see Fig. 3 for a chimpanzee atlas map; Vickery et al., 2020.)

### KIAA0319 and microstructure in posterior superior temporal cortex (area Tpt)

**Figure 4.**
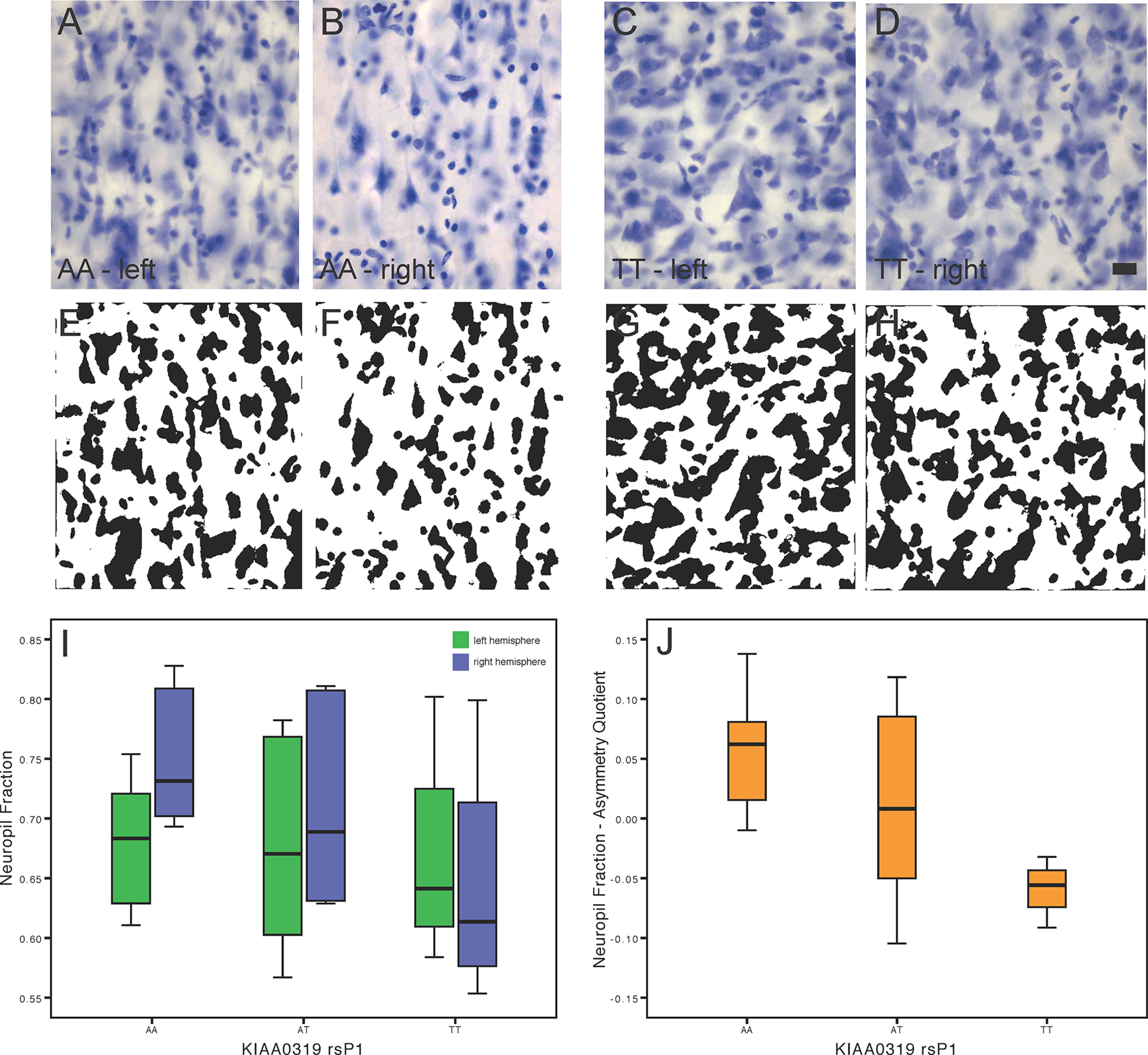
Hemispheric differences in neuropil fraction from posterior superior temporal cortex (area Tpt) of chimpanzees genotyped for *KIAA0319rsP1*. Photomicrographs of Nissl-stained sections from Layer III of area Tpt in an AA genotype chimpanzee’s left (***A***) and right (***B***) hemisphere, and a TT genotype chimpanzee’s left (***C***) and right (***D***) hemisphere. After background correction, the image frames are converted to binary for measurement of neuropil fraction (***E–H***) where the black pixels represent cell profiles and white pixels represent the neuropil space. Results from the sample are displayed as box and whisker plots of the distribution of neuropil fraction values between hemispheres (***I***) and the AQ (***J***) according to KIAA0319 rsP1 genotype.

We ran a series of multivariate general linear models with each dependent measure of cortical area Tpt microstructure in the left and right hemisphere with sex, age, and brain mass as covariates, and all three *KIAA0319* genotypes as fixed factors. Of these models, the only effect that approached a conventional statistical threshold was the *KIAA0319rsP1* genotype on right hemisphere neuropil fraction; *F*_(2,10)_ = 10.266, *p *=* *0.089) (see Table 2). The covariates sex, age, and brain mass did not have significant between-subjects effects. To further examine lateralization in cortical area Tpt microstructure, we also analyzed the AQ of each variable using general linear models with sex, age, and brain mass as covariates. Of these models, the only effect that approached a conventional statistical threshold was the *KIAA0319rsP1* genotype on AQ of neuropil fraction; *F*_(2,10)_ = 14.553, *p *=* *0.064. The covariates sex, age, and brain mass did not have significant between-subjects effects. Photomicrographs and results can be seen in Figure 4.

**Table 2:** Mean and AQ TpT total neuron, neuron density, volume, and neuropil space values (SE) for chimpanzees with different *KIAAo319rsP1* alleles

KIAA0319_rsP1	Genotype		
	AA	AT	TT
Average			
Total neuron	10118976	10931286	14375043
	(3616305)	(3275682)	(3531981)
Neuron density	29311	29926	37084
	(4392)	(3684)	(4852)
Volume	355	368	390
	(80)	(73)	(79)
Neuropil space	0.696	0.680	0.652
	(0.04)	(0.03)	(0.04)
Asymmetry (AQ)			
Total neuron	–0.409	–0.517	–0.263
	(0.31)	(0.28)	(0.31)
Neuron density	–0.225	–0.314	+0.132
	(0.19)	(0.16)	(0.21)
Volume	–0.16	–0.253	–0.352
	(0.31)	(0.28)	(0.30)
Neuropil space	0.066	0.010	–0.069
	(0.03)	(0.03)	(0.03)

## Discussion

Exploration of polymorphic variation in *KIAA0319* revealed a relatively variable coding sequence in chimpanzees. Across all four sub-species included in this study, a total of 29 SNVs were found spread across 12 of the 20 exons of the gene. Of these, a majority were nonsynonymous, thus causing amino acid changes, indicating low structural stability of the receptor protein in chimpanzees. Only a small proportion of SNVs were present in the flanking region (*N* = 4). This is in line with previous research showing that the most pronounced conservation across species in *KIAA0319* extends from the transcription start site to ∼1 kb upstream (Dennis et al., 2009). In humans, several SNVs in the upstream region of the gene have been shown to have strong associations with reading disorders and dyslexia (Cope et al., 2005; Harold et al., 2006; Dennis et al., 2009; Elbert et al., 2011) and similarly, in our study, the strongest association with brain phenotypic differences was found for variants *KIAA0319rsP1* and *KIAA0319rsP4*, despite a lack of overlap between human and chimpanzee promoter region SNVs. It thus appears for our chimpanzee population, that differential gene expression patterns are a likely driving factor behind this phenotypic variation, but further studies using gene expression assays are required to confirm this conclusion.

Findings from the SBM analysis showed that two SNVs in the promotor region of the *KIAA0319* gene (*KIAA0319rsP1* and *KIAA0319rsP4*), were associated with differences in gray matter volume of several brain regions. As hypothesized, these polymorphisms were associated with differences in gray matter covariation of the posterior superior temporal gyrus (including area Tpt and Heschl’s gyrus, HG). This brain region is associated with a variety of cognitive functions including language comprehension and production and previous studies have reported that atypical language processing is associated with SNVs in *KIAA0319* in humans (Hirayasu et al., 2000; Goulven and Tzourio-mazoyer, 2004; Dorsaint-Pierre et al., 2006; Warrier et al., 2009; Darki et al., 2012; Pinel et al., 2012; Jamadar et al., 2013; Friederici, 2015; Cardin et al., 2016; Eicher et al., 2016; Ozernov-Palchik and Gaab, 2016; Skeide et al., 2016).

The evidence that SNVs in *KIAA0319* are associated with variation in gray matter within the posterior superior temporal gyrus in chimpanzees is a somewhat paradoxical finding in light of the evidence of its role in language and reading, aptitudes that are arguably uniquely human. In fact, Dumas et al. (2021) recently reported that *KIAA0319* is under strong positive selection in humans compared with primate ancestors, and that this gene (and other communication-related genes) only diverged within the hominin lineage. At the most parsimonious level, the findings reported here suggest that although SNVs in *KIAA0319* are directly implicated in the morphology of the posterior superior temporal gyrus, their link to the mapping of linguistic functions onto these brain regions is less direct. In other words, SNVs in *KIAA0319* may be associated with both Tpt and HG morphology and specific kinds of language impairments, but the association is not causal, but rather mediating of other factors related to experience. Alternatively, the results reported here may indicate that there are more common neuropsychological processes that underlie individual variation in auditory-visual learning in human and nonhuman primates that are similarly influenced by SNV variation in *KIAA0319* in both taxa. For instance, studies on statistical learning have shown that human and nonhuman primates are sensitive to temporal regularities in auditory and visual signals, which some have argued is the foundation for speech as well as syntactical processing (Hauser et al., 2001; Newport et al., 2004; Petkov and Jarvis, 2012; Fagot, 2017; Heimbauer et al., 2018). In great apes, several studies have reported impressive human speech comprehension abilities, including the processing of sentences presented in English only (Savage-Rumbaugh et al., 1993; Sevcik and Savage-Rumbaugh, 1994; Brakke and Savage-Rumbaugh, 1995). Finally, neuroimaging and lesion studies have shown that area Tpt, HG, and a number of other regions are involved in processing of species-specific vocalizations (Heffner and Heffner, 1984; Poremba et al., 2003, 2004; Gil-da-Costa et al., 2006; Petkov et al., 2008; Taglialatela et al., 2009). Based on these collective findings, it is intriguing to speculate that SNVs in *KIAA0319* in nonhuman primates may underlie individual variation in auditory-visual learning and associated brain regions, although this hypothesis awaits additional research.

In addition, our microstructure analysis showed that *KIAA0319rsP1* variants are associated with hemispheric differences in neuropil fraction of chimpanzees. This suggests that *KIAA0319* expression may be involved in regulation of processes related to the formation and maintenance of synapses, dendrites, or axons in the microstructure of chimpanzee brains within a region directly involved in communication. Studies in rodents have shown that *KIAA0319* plays a role in neural migration during formation of the cerebral neocortex, the development of dendritic arbors and spines, axon outgrowth, and has functional impacts on auditory processing (Peschansky et al., 2010; Platt et al., 2013; Centanni et al., 2014; Franquinho et al., 2017; Guidi et al., 2017). However, there are limited data examining *KIAA0319* expression in the brains of humans or other primates (Kato et al., 2014; Muntané et al., 2017). It should be noted that our findings are based on a relatively small sample size, as is common for postmortem histologic studies. Nevertheless, in combination with the SBM results, these microstructure data further support the conclusion that *KIAA0319* influences neuroanatomy in a manner that is likely to be functionally significant. Future studies are needed to address whether the hemispheric differences in neuropil fraction are because of variation in synapses, dendritic morphology, or axon distributions.

## Synthesis

Reviewing Editor: Christine Portfors, Washington State University Decisions are customarily a result of the Reviewing Editor and the peer reviewers coming together and discussing their recommendations until a consensus is reached. When revisions are invited, a fact-based synthesis statement explaining their decision and outlining what is needed to prepare a revision will be listed below. The following reviewer(s) agreed to reveal their identity: NONE. Note: If this manuscript was transferred from JNeurosci and a decision was made to accept the manuscript without peer review, a brief statement to this effect will instead be what is listed below.

The reviewers agree that this is an interesting study with novel results. The manuscript would benefit from a more clear rationale and less use of abbreviations. A number of other concerns as noted below are necessary to improve the manuscript.

Summary of reviews:

Major concerns:

-One of the main findings of this paper, as outlined in the abstract, is the differences in brain microstructure measured using histology. At present this data is only summarized in a table. Some of the features presented can be abstract for a reader so it is critical that some images of the histology are shown. A suitable figure to add would include images of neuropil asymmetries (or lack thereof) for AA, AT, and TT alleles for rsP1.

-How were number of neurons calculated using just a Nissl stain? Cresyl violet also stains non-neuronal cells in the brain. A NeuN antibody, for example, would be more suitable for neuron-specific counts.

-The duplicate presentation of 3D renderings can be misleading. Combining figures 1 and 2, which includes (A) 3D renderings, (B) SBM scores for rsP1, and (C) SBM scores for rsP4 would facilitate a clearer presentation of the findings.

-It is unclear why 3D renderings of only component 1, 2, and 7 were shown. The methods say there were 8 components identified. More clarity is needed for why these were chosen or more discussion on the other 5 components.

-line 100: unclear, did this study generate new T1 data (as the methods would suggest) or used existing data as this sentence would imply. Needs clarification and citation for where the data came from, if applicable.

-line 248: it is unclear if new histology was generated for this study or if only used previously published (Spocter et al.) data, or both. As with the imaging data, this needs clarification.

-3D renderings of SBM components: At present, these images are not interpretable to someone not familiar with the organization of the non-human primate brain. eNeuro is a broad neuroscience journal and thus has diverse readership. It would help potential readers to include labels or a schematic for regions of interest.

Minor concerns:

-line 61: cite Pfenning et al., 2014 Science as it significantly contributed to knowledge of genes with hypothesized involvement in human speech

-line 131: how was the promoter region defined?

-line 132: why was the human genome used as a reference (both for mapping chimpanzee genomes and KIAA0319 reference) when chimpanzee has an annotated high quality reference genome? Mapping to the human genome assumes that the gene model is the same between the two species and may ignore species differences.

-line 272: please provide details on general linear models used

-line 332: rephrase, makes it seem like there’s only 12 exons in this gene (e.g. 29 SNVs found in 12 of the 20 exons of the gene)

-Discussion: Dumas et al., 2021 Genome Research recently found that KIAA0319 exhibits strong positive selection in humans. The discussion lines 357-366 make some hypotheses about the role of this gene in non-human primates, yet gene divergence may explain primate differences. Incorporating this new study into this discussion would be interesting.

-Figure legends: The figure legends are very brief which does not help with the interpretability of the figures. A figure legend typically begins with a one sentence title that summarizes what is being presented. The rest of the legend should include sufficient detail to interpret the figure in the absence of reading the results section. Composite figures like presented here should include letters to denote parts (e.g. Figure 1A, Figure 1B). Further detail will help readers navigate this manuscript.
